# Protective and immunostimulatory effects of in-feed preparations of an anticoccidial, a probiotic, a vitamin-selenium complex, and *Ferulago angulata* extract in broiler chickens infected with *Eimeria* species

**DOI:** 10.1186/s12917-021-03005-6

**Published:** 2021-09-15

**Authors:** Zahra Nooreh, Kamran Taherpour, Hossein Ali Ghasemi, Mohammad Akbari Gharaei, Hassan Shirzadi

**Affiliations:** 1 Department of Animal Science, Faculty of Agriculture, Ilam University, Ilam, Iran; 2 Department of Animal Science, Faculty of Agriculture and Natural Resources, Arak University, Arak, 38156-8-8349 Iran

**Keywords:** Broiler chickens, *Eimeria* challenge, *Ferulago angulata* extracts, Common feed additives, Growth performance, Immune response

## Abstract

**Background:**

Two experiments were conducted to compare the growth-promoting (experiment 1), protective, and immunostimulatory effects (experiment 2) of salinomycin, probiotic, a vitamin-selenium complex, and *Ferulago angulata* hydroalcoholic extract (FAE) against coccidiosis in broilers. In each experiment, 350 1-day-old broiler chickens were equally divided in 7 groups: uninfected negative control (NC); infected positive control (PC); or PC supplemented with salinomycin (Sal); probiotic (Pro); a combination of vitamin E, vitamin C, and selenium (ECSe); 200 mg/kg of FAE (FAE200); or 400 mg/kg of FAE (FAE400). All these groups (except NC) were challenged via oral gavage with oocysts of mixed *Eimeria* spp. on d 10 (experiment 1) or d 14 (experiment 2).

**Results:**

In the first trial, all treatments improved growth and feed conversion compared with the PC group, where the best values were noticed in the NC and FAE400 groups throughout the entire experimental period (d 1 to 42). Further, a lower mortality rate (*P* < 0.05) was observed in the NC, Sal, and FAE400 groups as compared to that in the PC group. In the second trial, intestinal lesion scores and total oocyst numbers were reduced in the Sal, Pro, and FAE400 groups compared to the PC group, albeit all coccidiosis-challenged groups had higher oocyst shedding (*P* < 0.05) compared to NC group. Immune responses revealed that among challenged birds, those fed diets Pro, ECSE, and FAE400 had significantly higher primary total and secondary total and IgG antibody titers against sheep red blood cells, serum and cecum specific IgG levels, and serum IFN-γ concentration than the PC group.

**Conclusions:**

Considering the results, dietary FAE, especially at high levels of inclusion in broiler diet (400 mg/kg), could beneficially influence growth performance and immune status under coccidiosis challenge, which was comparable to that of probiotic supplement.

## Background

As poultry production and meat consumption increase, greater importance is given to the quality of the poultry products. Coccidiosis is the most important parasite disease in the poultry industry, and causes great damage to poultry farmers. The disease is caused by apicomplexan protozoan parasites of the genus *Eimeria*, under which most common species include *acervulina*, *maxima*, and *tenella* [1]. Such parasites penetrate the intestinal epithelium in a site-specific manner causing the mucosal membrane and the underlying tissue to become inflamed and necrosed. The effects of damage can range from local destruction to systemic effects on the mucosal barrier and other tissues and can lead to death in more severe cases [2]. Although commercial broiler chickens are not currently being vaccinated against coccidiosis, new treatments urgently need to be developed that will provide adequate protection against various infections while also preventing performance adverse effects. In conventional management, coccidiosis is prevented and regulated by in-feed (or water) administration of anticoccidial supplements [3]. However, with increasing pressure to reduce (eliminate) the use of antibiotics and ionophore in poultry production, alternative strategies are required.

Feed additives that improve the integrity and function of the intestine, reduce inflammation or have a microbial modifying impact can complement vaccination, providing an alternative way to manage the disease in the antibiotic-free poultry production [4]. Thus, antibiotic alternatives, such as probiotics are becoming increasingly important in preventing and controlling infectious diseases including coccidiosis. Recent evidence revealed that various dietary probiotic supplements can influence host immunity against coccidiosis disease [5–7]. Some commercial probiotics have been also reported to enhance the development of both the intestinal lymphatic system and gastrointestinal epithelium [8, 9]. A healthy population of gut microbes would also be reported to support the inherent intestinal defense mechanisms, leading to better control of invading pathogens within the intestine [10].

On the other hand, it is suggested that the unbalanced oxidant/antioxidant status would probably be critical for the progression of the most parasitic diseases, especially coccidiosis [11, 12]. Therefore, substances that have antioxidant properties, such as tocopherol, ascorbic acid, selenium, and herbal extracts could be effective in protecting against coccidiosis. As it is well known, vitamins E, vitamin C, and selenium play a significant role in the antioxidant defense network, and can only work effectively in combination [13]. They have been reported to boost and modulate immune function under several disease challenges, including coccidiosis [14]. Colnago et al. [15] reported that broiler chickens challenged with 150,000 oocysts of *E. tenella* had higher body weight and survivability rate when fed the diet supplemented with 100 IU/kg of vitamin E or 0.25 mg/kg selenium compared with birds fed a non-supplemented diet, suggesting that Vitamin E and selenium boost the immune response after *Eimeria* immunization. Furthermore, although it is generally accepted that vitamin C can be synthesized by the bird, the pathological conditions, such as coccidiosis, may precipitate a need for exogenous sources of vitamin C by chickens [12].

The supplementation of phytogenic supplements consisting of herbal extracts has been also reported to reduce the coccidian oocyst shedding and intestinal lesions in broiler chickens [16–18]. *Ferulago angulata*, known as Chavir in Iran, is a plant of the *Apiaceae* family and is a native species of the western mountainous regions of Iran. This plant contains flavonoid and phenolic compounds that have a wide range of pharmacological effects, including antioxidant, anti-microbial, anti-inflammatory, and hemostatic activities [19, 20]. According to previous research, the dietary *F. angulata* could enhance growth performance and beneficially affect the intestinal microbial composition and antibody response of broiler chickens [21]. In this connection, Rafieian-kopaei et al. [22] reported that the addition of *Ferulago angulata* hydroalcoholic extract (FAE) to the diet could effectively increase antioxidant capacity and decrease plasma levels of malondialdehyde in rats. Although the antimicrobial activity of aerial parts of *F. angulata* against *Salmonella typhi*, *Staphylococcus aureus*, and *Listeria monocytogenes* have been confirmed [23], there is still a lack of literature on their use in the control of parasitic diseases such as coccidiosis.

Given all the above mentioned, the objective of this study was to assess and compare the potential growth-promoting, protective, and immunostimulatory effects of salinomycin, probiotics, vitamin-selenium complex, and FAE against coccidiosis in commercial broiler chickens.

## Results

### Experiment 1: growth parameters

The effects of experimental treatments on broiler performance during different growing periods are presented in Table 1. Dietary treatments did not significantly affect the ADG, ADFI, and FCR during d 1 to 10. From d 1 to 24, the ADG and FCR in all experimental groups, except ECSe and FAE200 groups, was also similar to that of the NC group, but better (*P* < 0.05) than the values in birds in the PC. Broiler chickens in the Pro and FAE400 groups have also exhibited a lower FCR (*P* < 0.05) than birds in PC, ECSe, and FAE200 groups during d 1 to 24. Over the entire experiment (d 1 to 42), all experimental groups improved (*P* < 0.05) ADG and FCR compared with the PC group, where the best values were recorded in the NC and FAE400 groups.

**Table 1:** Growth performance^1^ observed in broiler chickens infected with a mixture *of Eimeria* species at 10 d of age and provided with diets supplemented with salinomaycin (Sal), probiotic (Pro), combination of vitamin E, C, and selenium (ECSe), and *F. angulate* extracts at the levels of 200 and 400 m/kg (FAE200 and FAE400, respectively) in experiment 1

	Average daily gain (g)			Average daily feed intake (g)			Feed conversion ratio		
Treatments	d 1–10	d 1–24	d 1–42	d 1–10	d 1–24	d 1–42	d 1–10	d 1–24	d 1–42
NC^2^	19.84	39.34^a^	63.96^a^	26.38	60.58	115.6	1.33	1.54^bc^	1.81^d^
PC^3^	19.74	36.18^b^	54.58^d^	26.58	59.62	114.6	1.35	1.65^a^	2.10^a^
Sal + infected	20.18	38.90^a^	62.50^abc^	26.30	60.00	116.5	1.30	1.54^bc^	1.86^cd^
Pro + infected	20.36	38.87^a^	61.62^abc^	26.54	59.23	115.0	1.30	1.52^c^	1.86^cd^
ECSe + infected	19.62	37.11^ab^	59.94^bc^	26.18	59.70	116.2	1.33	1.61^ab^	1.94^bc^
FAE200 + infected	20.15	37.12^ab^	58.87^c^	26.14	59.77	117.4	1.30	1.61^ab^	1.99^b^
FAE400 + infected	20.35	39.15^a^	62.99^ab^	26.02	59.13	115.3	1.28	1.51^c^	1.83^d^
SEM^4^	0.43	1.08	1.72	0.39	1.74	2.24	0.027	0.032	0.045
*P*-value	0.105	0.019	0.005	0.524	0.886	0.746	0.170	0.008	0.013

^a-c^Means within a column not sharing the same superscript are different at *P* < 0.05

^1^Values are means of 5 pens per treatment combination with 10 male broiler chickens (*n* = 5 per treatment)

^2^Negative control (not treated and uninfected)

^3^Positive control (not treated, but infected)

^4^Pooled standard error of mean

**Fig. 1:**
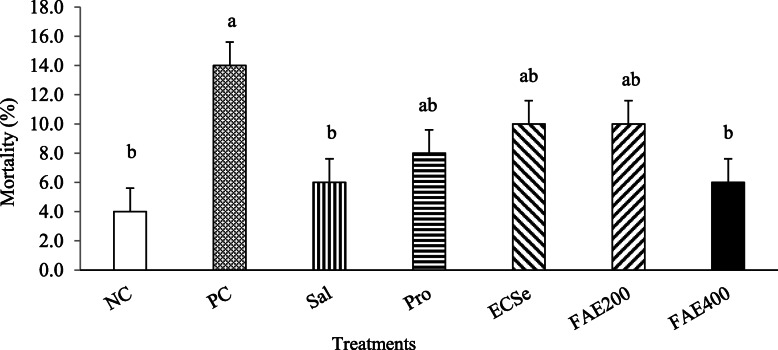
Mortality rate (d 1 to 42) observed in broiler chickens infected with a mixture of *Eimeria* species at 10 d of age in experiment 1. Mortality was converted to a square root arcsine transformation prior to analysis. Values with different superscripts are significantly different by Tukey’s pairwise test (*P* < 0.05). Each bar represents the mean values and standard errors representing 5 replicates per treatment and 10 birds per replicate pen (*n* = 50 per treatment). The experimental groups were: NC, negative control (non-treated, uninfected); PC, positive control (non-treated, infected); SAL, salinomycin supplemented diet, infected (60 mg/kg of diet); Pro, probiotic supplemented diet, infected (1 g/kg of diet); ECSe, vitamin-selenium supplemented diet, infected (a combination of 200 IU vitamin E, 250 mg vitamin C, and 0.2 mg selenium per kg of diet); FAE200, *Ferulago angulate* extract supplemented diet, infected (200 mg/kg of diet); FAE400, *Ferulago angulate* extract supplemented diet, infected (400 mg/kg of diet)

As Fig. 1 shows, the overall mortality rate (d 1 to 42) was also lower (*P* < 0.05) for birds in the NC, Sal, and FAE400 groups than the PC group.

### Experiment 2: lesion score, oocyst output, and immune responses

#### Intestinal lesion score

The results of the coccidiosis lesion score (on 7th days post-inoculation) are presented in Table 2. The intestinal lesions in the duodenum (the site of *E. acervulina* infection) and cecum (the site of *E. tenella* infection) were lower (*P* < 0.05) for the birds that received the all treatments as compared with that of PC group. By comparison, the duodenal and cecal lesion scores of broilers in the Sal and FAE400 groups was similar (*P* > 0.05) to that of those in the NC group, but lower (*P* < 0.05) than that of those in the ECSe and FAE200 groups. In the Jejunum, lesion score caused by *E. maximain* infection in broilers fed any of the diets, except diet FAE200, was lower (*P* < 0.05) than that of those in the PC group, but higher (*P* < 0.05) than those in the NC group.

**Table 2:** Intestinal lesion scores^1^ observed in broiler chickens 6 d after mixed *Eimeria* species challenge at 14 d of age and provided with diets supplemented with salinomaycin (Sal), probiotic (Pro), combination of vitamin E, C, and selenium (ECSe), and F. angulate extracts at the levels of 200 and 400 m/kg (FAE200 and FAE400, respectively) in experiment 2

Treatments	Duodenum	Jejunum	Cecum
NC^2^	0.00^d^	0.00^c^	0.00^d^
PC^3^	3.00^a^	2.60^a^	2.20^a^
Sal + infected	0.60^cd^	1.00^b^	0.20^cd^
Pro + infected	1.00^c^	1.00^b^	0.40^cd^
ECSe + infected	2.00^b^	1.60^b^	1.20^b^
FAE200 + infected	2.00^b^	1.80^ab^	1.40^b^
FAE400 + infected	0.60^cd^	1.00^b^	0.40^cd^
SEM^4^	0.197	0.218	0.163
*P*-value	< 0.001	< 0.001	< 0.001

^a-c^Means within a column not sharing the same superscript are different at *P* < 0.05

^1^Values are means of 10 cages per treatment combination with 2 male broiler chickens (*n* = 10 per treatment). The average of 2 birds per replicate cage was applied for statistical analysis of these variables

^2^Negative control (not treated and uninfected)

^3^Positive control (not treated, but infected)

^4^Pooled standard error of mean

#### Excreta oocyst shedding

**Fig. 2:**
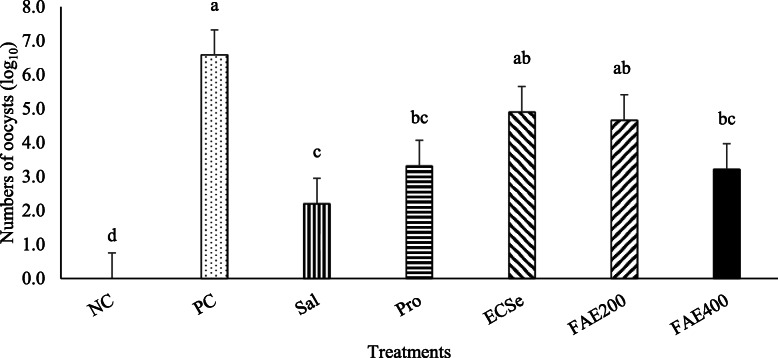
Oocysts per gram of droppings observed in broiler chickens (d 20–24) after mixed Eimeria species challenge at 14 d of age in experiment 2. Values with different superscripts are significantly different by Tukey’s pairwise test (*P* < 0.05). Each bar represents the mean values and standard errors representing 10 replicates (cages) per treatment (*n* = 10 per treatment). The experimental groups were: NC, negative control (non-treated, uninfected); PC, positive control (non-treated, infected); SAL, salinomycin supplemented diet, infected (60 mg/kg of diet); Pro, probiotic supplemented diet, infected (1 g/kg of diet); ECSe, vitamin-selenium supplemented diet, infected (a combination of 200 IU vitamin E, 250 mg vitamin C, and 0.2 mg selenium per kg of diet); FAE200, *Ferulago angulate* extract supplemented diet, infected (200 mg/kg of diet); FAE400, *Ferulago angulate* extract supplemented diet, infected (400 mg/kg of diet)

Total oocysts per gram of excreta on d 20 to 24 from each pen as presented in Fig. 2. Excreta oocyst shedding decreased (*P* < 0.05) in all of the treatment groups, except ECSe and FAE200 treatments, compared to the PC group. However, the NC group showed fewer oocyst shedding (*P* < 0.05) than all treatment groups following the coccidiosis infection.

#### Humoral immune response

The effects of dietary treatments on antibody-mediated immune response against SRBC in broiler chickens are detailed in Table 3. Birds receiving any of the test additives, except 200 mg/kg of FAE, had similar (*P* > 0.05) total antibody titer for the primary response, as well as total and IgG antibody titers for the secondary response, as those in the NC group, but higher (*P* < 0.05) than those in the PC group. In addition, the birds receiving the Sal, Pro, and FAE400 diets were similar with a higher (*P* < 0.05) response for IgG antibody titer during the secondary challenge when compared with the PC and FAE200 treatments.

**Table 3:** Antibody response to sheep red blood cells (SRBC; log_2_)^1^ observed in broiler chickens infected with a mixture of *Eimeria* species at 14 d of age and provided with diets supplemented with salinomaycin (Sal), probiotic (Pro), combination of vitamin E, C, and selenium (ECSe), and F. angulate extracts at the levels of 200 and 400 m/kg (FAE200 and FAE400, respectively) in experiment 2

	Primary anti-SRBC antibody response (d 14)			Secondary anti-SRBC antibody response (d 21)		
Treatments	IgG	IgM	Total antibody	IgG	IgM	Total antibody
NC^2^	1.64	1.91	3.55^ab^	4.61^ab^	3.63	8.24^a^
PC^3^	1.23	1.20	2.43^c^	2.80^c^	3.02	5.82^b^
Sal + infected	1.61	2.01	3.62^ab^	4.75^a^	3.22	7.97^a^
Pro + infected	1.64	2.19	3.83^a^	5.20^a^	3.01	8.21^a^
ECSe + infected	1.68	1.93	3.61^ab^	4.75^a^	3.24	7.99^a^
FAE200 + infected	1.40	1.44	2.84^bc^	3.62^bc^	3.58	7.20^ab^
FAE400 + infected	1.56	2.02	3.58^ab^	4.84^a^	3.54	8.38^a^
SEM^4^	0.286	0.411	0.330	0.347	0.404	0.569
*P*-value	0.711	0.089	0.040	0.001	0.521	0.009

^a-c^Means within a column not sharing the same superscript are different at *P* < 0.05

^1^Values are means of 10 cages per treatment combination with 2 male broiler chickens (*n* = 10 per treatment). The average of 2 birds per replicate cage was applied for statistical analysis of these variables

^2^Negative control (not treated and uninfected)

^3^Positive control (not treated, but infected)

^4^Pooled standard error of mean

**Fig. 3:**
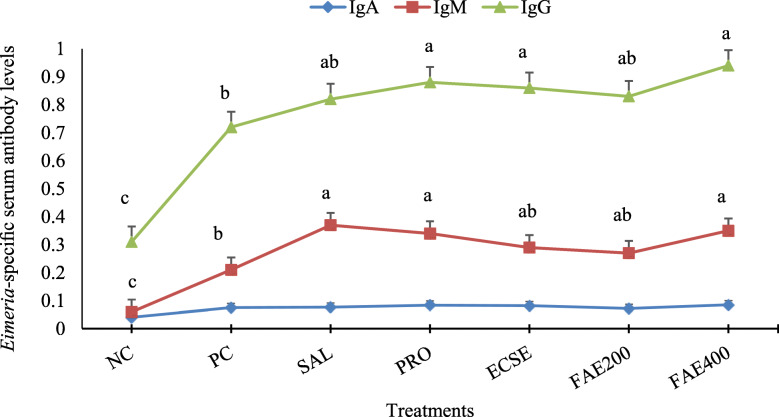
ELISA determination of specific IgA, IgM, and IgG antibodies against recombinant coccidia 3-1E antigen (absorbance at 450 nm) in the serum at 24 d of age observed in broiler chickens infected with a mixture of *Eimeria* species at 14 d of age in experiment 2. Means with different superscripts are significantly different by Tukey’s pairwise test (*P* < 0.05). Values are means ± SEM, representing 10 replicates per treatment and 2 birds per replicate cage (*n* = 10 per treatment). The average of 2 birds per replicate cage was applied for statistical analysis of these variables. The experimental groups were: NC, negative control (non-treated, uninfected); PC, positive control (non-treated, infected); SAL, salinomycin supplemented diet, infected (60 mg/kg of diet); Pro, probiotic supplemented diet, infected (1 g/kg of diet); ECSe, vitamin-selenium supplemented diet, infected (a combination of 200 IU vitamin E, 250 mg vitamin C, and 0.2 mg selenium per kg of diet); FAE200, *Ferulago angulate* extract supplemented diet, infected (200 mg/kg of diet); FAE400, *Ferulago angulate* extract supplemented diet, infected (400 mg/kg of diet)

The levels of serum *Eimeria* antigen-binding specific antibody levels measured at 24 d (10 d postinfection) are shown in Fig. 3. The birds infected with *Eimeria* oocysts (PC group) had greater IgM and IgG levels (*P* < 0.05) than uninfected birds (NC group). Among infected birds, serum specific IgM level of the Sal, Pro, and FAE400 groups were higher (*P* < 0.05) compared with the PC group. Specific IgG level of the groups fed any of the diets, except diets Sal and FAE200, were higher (*P* < 0.05) compared with that of the PC group.

**Fig. 4:**
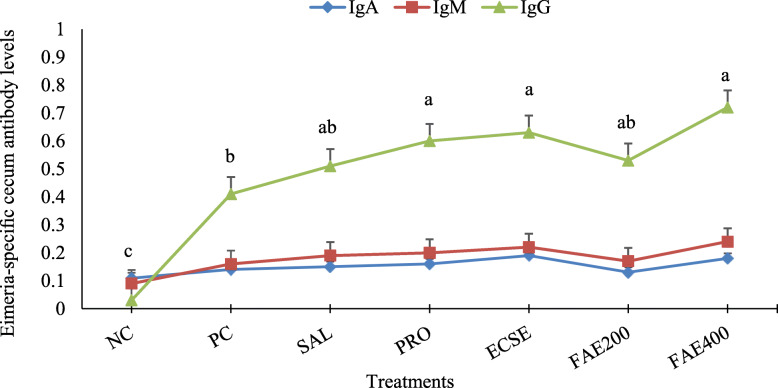
ELISA determination of specific IgA, IgM, and IgG antibodies against recombinant coccidia 3-1E antigen (absorbance at 450 nm) in the caeca at 24 d of age observed in broiler chickens infected with a mixture of *Eimeria* species at 14 d of age in experiment 2. Means with different superscripts are significantly different by Tukey’s pairwise test (*P* < 0.05). Values are means ± SEM, representing 10 replicates per treatment and 2 birds per replicate cage (*n* = 10 per treatment). The average of 2 birds per replicate cage was applied for statistical analysis of these variables. The experimental groups were: NC, negative control (non-treated, uninfected); PC, positive control (non-treated, infected); SAL, salinomycin supplemented diet, infected (60 mg/kg of diet); Pro, probiotic supplemented diet, infected (1 g/kg of diet); ECSe, vitamin-selenium supplemented diet, infected (a combination of 200 IU vitamin E, 250 mg vitamin C, and 0.2 mg selenium per kg of diet); FAE200, *Ferulago angulate* extract supplemented diet, infected (200 mg/kg of diet); FAE400, *Ferulago angulate* extract supplemented diet, infected (400 mg/kg of diet). Means with different letters differ significantly (*P* < 0.05)

As shown in Fig. 4, all infected broiler chickens had higher specific IgG level (*P* < 0.05) versus the uninfected chickens (NC group). The specific IgG level in the Pro, ECSe, and FAE400 groups were higher (*P* < 0.05) compared with the control infection group (PC group) at 10 d postinfection, whereas IgA and IgM were not different (*P* > 0.05).

#### Cellular immune response

**Fig. 5:**
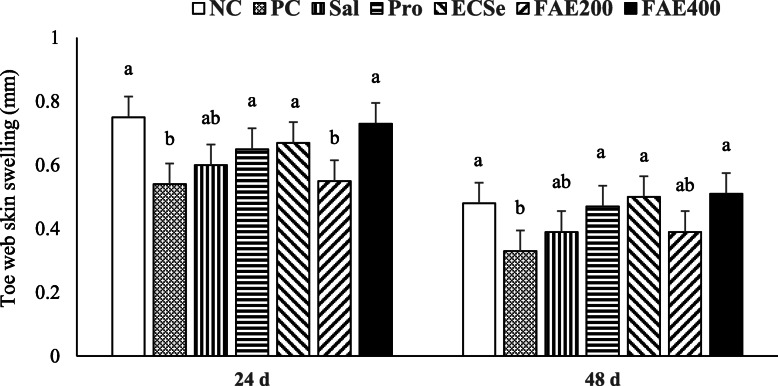
Lymphoproliferative response 24 and 48 h after intradermal injection of phytohemagglutinin-P at 20 d of age observed in broiler chickens infected with a mixture of *Eimeria* species at 14 d of age in experiment 2. Values with different superscripts are significantly different by Tukey’s pairwise test (*P* < 0.05). Each bar represents the mean values and standard errors representing 10 replicates (cages) per treatment and 2 birds per replicate cage (*n* = 10 per treatment). The average of 2 birds per replicate cage was applied for statistical analysis of these variables. The experimental groups were: NC, negative control (non-treated, uninfected); PC, positive control (non-treated, infected); SAL, salinomycin supplemented diet, infected (60 mg/kg of diet); Pro, probiotic supplemented diet, infected (1 g/kg of diet); ECSe, vitamin-selenium supplemented diet, infected (a combination of 200 IU vitamin E, 250 mg vitamin C, and 0.2 mg selenium per kg of diet); FAE200, *Ferulago angulate* extract supplemented diet, infected (200 mg/kg of diet); FAE400, *Ferulago angulate* extract supplemented diet, infected (400 mg/kg of diet)

As Fig. 5 shows, the relative increases in toe web swelling were found to be higher (*P* < 0.05) in broiler chickens in the NC, Pro, ECSe, and FAE400 groups compared with the PC group at both 24 and 48 h post PHA-P injection.

**Fig. 6:**
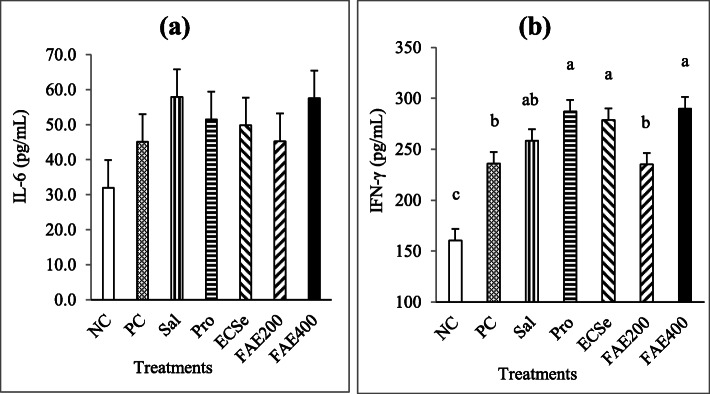
Serum concentrations of IL-6 (**a**) and IFN-γ (**b**) at 24 d of age observed in broiler chickens infected with a mixture of *Eimeria* species at 14 d of age in experiment 2. Values with different superscripts are significantly different by Tukey’s pairwise test (*P* < 0.05). Each bar represents the mean values and standard errors representing 10 replicates per treatment and 2 birds per replicate cage (*n* = 10 per treatment). The average of 2 birds per replicate cage was applied for statistical analysis of these variables. The experimental groups were: NC, negative control (non-treated, uninfected); PC, positive control (non-treated, infected); SAL, salinomycin supplemented diet, infected (60 mg/kg of diet); Pro, probiotic supplemented diet, infected (1 g/kg of diet); ECSe, vitamin-selenium supplemented diet, infected (a combination of 200 IU vitamin E, 250 mg vitamin C, and 0.2 mg selenium per kg of diet); FAE200, *Ferulago angulate* extract supplemented diet, infected (200 mg/kg of diet); FAE400, *Ferulago angulate* extract supplemented diet, infected (400 mg/kg of diet)

As shown in Fig. 6a, the serum concentration of IL-6 between the different experimental groups was not different (*P* > 0.05) at 10 d postinfection (d 24). In contrast, the birds infected with *Eimeria* oocysts had higher (*P* < 0.05) serum IFN-γ concentration than NC birds (Fig. 6b). The serum IFN-γ concentration in broiler chickens fed with the Pro, ECSe, and FAE400 diets were found significantly higher than the PC group.

## Discussion

The current study evaluated the protective effect of a probiotic, a vitamin-selenium complex, and FAE as feed additives against the coccidiosis challenge compared to salinomycin. The most obvious symptom of poultry coccidiosis is growth retardation, which is described by weight loss, and this has a major economic impact on the poultry industry [24]. On the other hand, the damage to the intestinal tissue decreases nutrient absorption and thus reduces feed efficiency and body weight, and eventually results in poor growth performance [25]. The results of the current study showed that the overall BWG and FCR of the broilers treated with probiotic and FAE (at a high level) was significantly better than the challenged chickens that did not receive any additive but similar to the chickens that were infected and had received salinomycin.

In line with the results of the current experiment, Giannenas et al. [8] reported that adding probiotics to the diet of the broilers challenged with coccidiosis could minimize its negative effects on growth performance. Probiotic strains are able to compete for binding sites and occupy the most common receptors on the mucus of the gut wall. This could decrease penetration by motile stages of *Eimeria* parasites, and subsequently, their colonization and shedding [9]. Therefore, the reason for improving the BWG and FCR in probiotic treatment may be related to its effect on the microbial population of the gastrointestinal tract, in which case better bird growth will be provided by balancing the microbial population and protective effects against the pathological consequences of coccidiosis.

In this study, although the dietary supplementation with a combination of vitamin E, vitamin C, and selenium had a positive effect on the growth performance of the challenged broilers, they did not show the same growth performance as those of the NC group. In line with the results of this study, improvements in body weight gain and feed efficiency were observed in coccidiosis-challenged broiler chickens fed a diet supplemented with with selenium [14, 15], vitamin E [12, 26], or vitamin C [12]. A possible explanation is the well-documented immunomodulatory effects of selenium [26], vitamin E [27], and vitamin C [28], which boosts the birds’ immune responses against *Eimeria*, resulting in the positive effects of ECSe treatment against the coccidiosis.

In the current experiment, there were significant positive effects of a high level of FAE on ADG and FCR, which was similar to those observed in the Sal and Pro groups. In addition, among the challenged treatments, the Sal and FAE400 groups had the lowest mortality rate, which was comparable to that in the NC group. Previous studies have suggested herbal extracts, especially bioactive compounds like polyphenols, may have potential as alternative therapeutic agents against coccidiosis [29, 30], but no studies have examined the effect of FAE. It is well known that phenolic compounds such as thymol and carvacrol cause degradation of cell membrane lipids in the presence of molecular oxygen, leading to changes in a membrane permeability to ions, which is accompanied by cell death as a result of an inhibition of cellular enzyme discharge [31]. Natural bioactive compounds such as flavonoids, tannins, and saponins have also displayed high antioxidant and anti-inflammatory activities [32]. This unique complex of bioactive constituents can protect the intestinal epithelium from oxidative damage, which can cause inflammation [33, 34]. Furthermore, tannins are well-known for their anti-parasitic and antimicrobial properties because of their ability to complex with a large variety of metal ions and microbial [18, 35, 36]. The better ADG and FCR and lower mortality rate in coccidiosis-challenged broilers fed the high-FAE diet supported the above-mentioned results.

*Eimeria* is an intracellular parasite that should invade the host cells for reproduction, and in this case, the parasite should first adhere to the epithelial cells. Fewer and less severe levels of lesions indicate less disruption to the epithelium of the intestine, leading to a greater chance of disease recovery for infected birds [3]. In this study, birds receiving probiotic, vitamin-selenium mixture, and a high dose (400 mg/kg) of FEA in the diet exhibited the lowest lesion score and shed fewer oocysts in the excreta as compared with the PC birds. The capacity of probiotic bacteria to adhere and compete for adhesion sites in the intestinal epithelium and the capacity to produce organic acids and effective antibacterial compounds might result in the impairment of *Eimeria* colonization in the gut. Corroborating our findings, Ritzi et al. [6] found that broiler provided a probiotic mixture in the feed shed significantly fewer *Eimeria* oocysts compared with the challenged control. Dominguez et al. [12] also reported that arginine, vitamin E and C supplemented together in a broiler diet following the challenge with coccidia vaccine led to the decreased lesion scores within the jejunum and cecum compared with the challenged control group. Interestingly, in this study, broiler chickens receiving the FAE400 diet handled the challenge during the infection period better than birds treated with other feed additives with regard to the mean lesion score and oocyst output, which was comparable to the birds fed salinomycin, an in-feed anticoccidial. However, no consistent results were observed in studies of the efficiency of herbal products on oocyst shedding and lesion scores in broiler chickens. Oregano oil supplemented at 300 or 600 ppm in the diet of broilers challenged with *E. acervulina* and *E. maxima* showed a reduction in lesion score without affecting the oocysts [37]. Duffy et al. [38] also recorded a significant reduction in the average cecal lesion score in the broiler chickens fed plant-derived product Natustat or salinomycin compared to the challenged control birds on d 21. In another study, extracts of *Tulbaghia violacea* as an herbal plant, added in the feed of *Eimeria*-infected broilers exhibited a decrease in oocysts production [16]. In contrast, Scheurer et al. [33] examined the effects of different dietary phytogenic products on avian coccidiosis and showed that none of the phytogenic products was effective at the tested dosage in reducing intestinal lesion scores and oocyst shedding in coccidiosis-challenged broilers. Some of the factors associated with the result variation due to herbal extracts could include volatility and stability of the compound, composition, purity, quality, and methods of extraction.

The results of the present study also showed that coccidiosis challenge significantly decreased primary total anti-SRBC titers, as well as total and IgG antibody responses to SRBC after secondary immunization. In contrast, the levels of systemic and cecum mucosal *Eimeria* antigen-binding antibodies, as well as serum IFN-γ concentration, in broiler chickens infected with mixed species of *Eimeria* were significantly greater than those of uninfected birds measured at 10 d postinfection (24 d). Humoral-mediated immunity is activated by infection with *Eimeria* [39], and antibody levels have been shown to be linked to the extent of the infection and the level of parasite exposure [40]. Antibodies have been suggested to minimize the invasion of some *Eimeria* species when parasites come into direct contact with local antibodies before entering the host [41]. Some recent studies have shown a decreased antibody response following the coccidiosis challenge [42–44]. With regard to the results of antibody responses against SRBC and serum specific IgG levels, our data suggest that all dietary supplements, except 200 mg/kg *F. angulate* extract, have the potential to positively affect immune function in broiler chickens challenged with coccidiosis. In addition, the Pro, ECSe, and FAE400 groups exhibited higher production of *Eimeria* antigen-binding specific IgG in the serum and cecum, as well as serum IFN-γ concentration and lymphoproliferative response (measured as a toe-web swelling 24 and 48 h after PHA-P injection), than the PC group.

Similar results have been demonstrated in coccidiosis-vaccinated broilers in which administration of lactic acid-producing bacteria, including *L. reuteri*, *Bifidobacterium animalis*, *Enterococcus faecium*, and *Pediococcus acidilactici* (1 × 10^8^ cfu/bird per day) could modulate the immune response to the vaccine [45]. Administration of a probiotic containing a blend of *Saccharomyces* and *Pediococcus* has shown an improvement over the antibody response in Eimeria-challenged broilers [3]. Recent evidence indicates that probiotics may enhance host defenses against infection because of the bacteria’s effect on host immunity and gut integrity under enteric pathogen challenge [7, 9, 46].

It has also been stated that vitamin E, vitamin C, and selenium as the potent antioxidant compounds in biological systems possess immunomodulatory effects [27, 28, 47, 48]. Another study reported that simultaneous supplementation of arginine, vitamin E, and vitamin C in coccidiosis-challenged broilers resulted in higher nitric oxide concentration and glutathione peroxidase activity, as well as a stronger innate immune response [12]. In this regard, it seems that birds fed the vitamin-selenium mixture might be better prepared to an *Eimeria*-causing infection in the field.

Our results also indicate that supplementation of 400 mg/kg FAE in the broiler diet could result in enhancement of resistance to coccidiosis, probably by enhancing humoral immune responses against *Eimeria* species in coccidia-challenged broilers. Limited research exists on the impacts of *Ferulago angulata* and their extracts on poultry health-related parameters during coccidiosis. Previous report indicates that the use of 8 g/kg *F. angulate* powder in the broiler diet may promote humoral immunity by increasing serum total anti-SRBC titers [21]. In another study in broiler chickens, a blend of carvacrol, cinnamaldehyde, and capsicum oleoresin was useful in promoting cell-mediated immunity leading to an increase in natural killer T cells, CD4+ (helper) T cells, CD8+ (cytotoxic) T cells, macrophages, and cytokines such as Interleukins-6 and interferon-γ that enhanced the host immunity against coccidiosis by stimulating both the innate and the adaptive (humoral) immune response [11]. Recent studies also suggest that dietary supplementation with herbal products, especially polyphenolic bioactive molecules, beneficially modulates intestinal microbiota, even in the presence of a coccidiosis challenge [49, 50]. A well-balanced gut microbiotais reported to be directly responsible for an improvement in intestinal health via the antagonization of pathogenic bacteria, and modulation of immunity [51, 52]. Therefore, positive changes in the intestinal microflora caused by FAE treatment may be directly related to the best broiler immune function, but how this relationship may influence the resistance to coccidiosis is not clear.

## Conclusion

In conclusion, high FAE dose helped the coccidiosis-challenged broiler chickens to perform as well as the chickens receiving probiotic and salinomycin, and better than chickens in the negative control. The anticoccidial efficacy of salinomycin, probiotic, and high dose (400 mg/kg) of FAE were more pronounced than any other supplement in terms of lowering intestinal lesion and oocyst output, after a mixed *Eimeria* challenge at 14 d of age. Moreover, feeding Pro, ECSe, and FAE400 diets could positively influence humoral and cellular immunity, which may be one of the mechanisms through which they exert beneficial effects on intestinal health in broiler chickens. However, further studies are required to clarify whether the restorative effect of these treatments was due to a specific mode of action against *Eimeria* parasite or immunomodulatory activities.

## Methods

The current study was carried out in compliance with the ARRIVE guidelines. The procedures involving animal care and use were approved by Animal Ethics Committee of Ilam University (approval number 96–212). The experimental birds received humane care and every effort was made to minimize pain and discomfort to the birds (by inspecting animals, housing and husbandry of animal, as well as reviewing records and reports) as outlined in the Guide for the Care and Use of Experimental Animals by Iranian Council of Animal Care [53].

### Anticoccidial, probiotic, and *Ferulago angulate* extract

The salinomaycin (Sacox 60, Intervet/Merck; Millsboro, DE), as an ionophore anticoccidial agent, was included at a rate of 60 mg/kg in all dietary phases for appropriate treatments. The multi-strain probiotic (Primalac, Star Labs Inc., Clarksdale, MO) containing *L. casei*, *L. acidophilus*, *Bifidobacterium bifidum*, *Streptococcus faecium* and *Aspergillus oryzae* was added at a dietary level of 1 g/kg. This level of probiotic supplementation was selected to warrant the survival and establishment in the intestines of treated broilers.

*Ferulago angulata* plants were collected from Sirvan Mountains (Ilam province, Iran). After botanical authentication by Dr. Valiollah Mozaffarian, Professor of Botany, Research Institute of Forests and Rangelands (Tehran, Iran), a voucher specimen was deposited at Herbarium of Ilam University Medicinal and Aromatic Plants Research institute (Herbarium number IURS-1106). We received prior permission from the Forests Range and Watershed Management Organization of Ilam Province, and no endangered or protected species were sampled. The aerial parts of the plant (flower and leaf) were washed with clean water, air-dried and then ground into a fine powder. Next, the dry powder was macerated at room temperature in ethanol (70:30 ethanol:water, v/v) for 72 h. The resulting extract was filtered through Whatman filter paper No. 2, and the ethanol extract of *F. angulata* was evaporated under reduced pressure to remove ethanol and the final product was obtained as a powder state (32% yield) after lyophilization [54]. Gas chromatography–mass spectrometry (GC 7890, Agilent Co., USA, equipped with a MS 5975C detector and HP-5 ms capillary column) analyses were carried out to detect the components of FAE. The initial column temperature was set at 40 °C lasting 3 min, and then programmed at 295 °C for 10 min, with a heating rate of 10 °C/min. The total analysis time was about 38 min. The main active components of FAE are shown in Table 4.

**Table 4:** Major phytocomponents identified by GC-MS in the hydroalcoholic extract of *Ferulago angulata*

Peak No.	Compounds	RT	Yield (g/kg)
1	Phenol	14.91	9.7
2	Pyrone	17.26	7.2
3	Malic acid	17.83	29.7
4	5-Hydroxymethylfurfural	18.59	46.2
5	Thymol	19.28	24.2
6	Carvacrol	19.65	53.2
7	Benzene, [2-nitro-1-(4-pentenylthio) ethyl]-	20.08	18.3
8	Dodecanoic acid, methyl ester	21.42	11.9
9	Benzocyclooctene, 7,8-dimethyl-	22.16	12.8
10	1,2,3-Benzenetriol (phenol)	22.40	58.4
11	beta.-D-Glucopyranose, 1,6-anhydro-	23.34	20.8
12	Myristic acid, methyl ester	23.75	17.4
13	Butyraldehyde, semicarbazone	25.07	57.9
14	9-Hexadecenoic acid, methyl ester	25.80	11.6
15	Pentadecanoic acid, 14-methyl-, methyl ester	25.98	55.6
16	Hexadecanoic acid, ethyl ester	26.49	44.2
17	9-Octadecenoic acid, methyl ester	27.86	207.5
18	Methyl stearate	28.02	29.1
19	2H-Pyrane-5-carboxamide (heterocyclic compound)	28.34	67.4
20	9,12,15-Octadecatrienoic acid, ethyl ester	28.57	37.2
21	Benzenesulfonothioic acid (thiosulfonic acid)	28.69	66.1
22	1H-1,3-Benzimidazole (heterocyclic compound)	29.20	30.2
23	2-(2-Nitrovinyl) furan	30.38	17.5

*RT* Retention time

### Experimental design

The study was divided into two experiments; experiment 1 was meant for the evaluation of growth performance; whereas, experiment 2 was meant for the evaluation of the protective and immunostimulatory efficacy of salinomycin, probiotic, vitamin-selenium complex, and FAE in broiler chickens when challenged with coccidiosis. A total of 700 male broiler chickens (Ross 308, Aviagen Inc., Huntsville, AL, USA) were obtained from a local commercial hatchery (Morghparvar Ltd., Arak, Markazi Province, Iran). On d 0, birds were housed in two independent rooms, one equipped with floor pens (experiment 1, *n* = 350) and the other equipped with wire cages (experiment 2, *n* = 350). In both experiments, broiler chickens were fed a starter diet from d 0 to 10 and a grower diet from d 11 to 24. A finisher diet was also provided from day 24 until the end of the experiment (d 42) for experiment 1. The non-medicated diets (Table 5) were formulated according to the nutritional recommendation by Ross 308 (2014) and fed in mash form. The experimental groups for both experiments were: 1) negative control (NC; commercial feed, no additives, untreated, and uninfected); 2) positive control (PC; commercial feed, no additives, untreated, infected with a mixture of *Eimeria* species); 3) PC + 60 mg/kg salinomycin (Sal); 4) PC + 1 g/kg probiotic PrimaLac (Pro); 5) PC + a vitamin-selenium complex preparation (ECSe; 200 IU vitamin E/kg feed + 250 mg vitamin C/kg feed + 0.2 mg selenium/kg feed); 6) PC + 200 mg/kg FAE (FAE200), and 7) PC + 400 mg/kg FAE (FAE400). Both PC and NC birds were fed a standard corn-soybean meal-based diet (Table 5) without additives, anticoccidial, and growth promoters.

**Table 5:** Composition of the basal starter, grower, and finisher diets and their nutrient profile

Item	Starter (1–10 d)	Growth (11–24 d)	Finisher (25–42 d)
Ingredient, %			
Corn	47.03	59.60	65.99
Wheat	5.58	5.00	5.00
Soybean meal (44% crude protein)	29.02	16.15	10.28
Corn gluten meal (60% crude protein)	10.00	11.48	11.50
Soybean oil	3.50	3.40	3.09
Limestone	1.45	1.23	1.00
Dicalcium phosphate	1.95	1.80	1.83
Sodium chloride	0.20	0.20	0.20
Vitamin premix^a^	0.25	0.25	0.25
Mineral premix^b^	0.25	0.25	0.25
DL-Methionine	0.52	0.58	0.57
L-Lysine HCl	0.25	0.06	0.04
Calculated value			
Metabolizable energy (kcal/kg)	2950	3000	3050
Crude protein (%)	22.00	20.00	19.00
Lysine (%)	1.30	1.20	1.10
Methionine (%)	0.56	0.54	0.52
Methionine + cysteine (%)	0.92	0.90	0.88
Calcium (%)	1.04	0. 95	0.92
Available phosphorus	0.52	0.44	0.42

^a^Provided per kilogram of diet: trans-retinol, 9000 IU; cholecalciferol, 2500 IU; α-tocopherol acetate, 45 mg; vitamin K, 5 mg; vitamin B1, 2 mg; vitamin B2, 6 mg; vitamin B6, 5 mg; vitamin B12, 0.03 mg; nicotineamide, 30 mg; pantothenic acid, 15 mg; folic acid, 1.1 mg; biotin, 0.13 mg; and choline, 450 mg

^b^Provided per kilogram of diet: Mn, 100 mg; Fe, 80 mg; Zn, 100 mg; Cu, 10 mg; I, 0.5 mg; Co, 0.2 mg; Se, 0.15 mg

The room temperature was set at 34 °C on the day of arrival, and then reduced by 0.40 °C per day until 24 °C where it remained for the rest of the trial. The environmental relative humidity was maintained at 50–65% by periodical spraying the walkways with water and adjusting the humidifiers. The lighting program used was 24 L∶0D from d 0 to 3 and 23 L∶1D for the remainder of the experiment.

### Coccidia species challenge

**Fig. 7:**
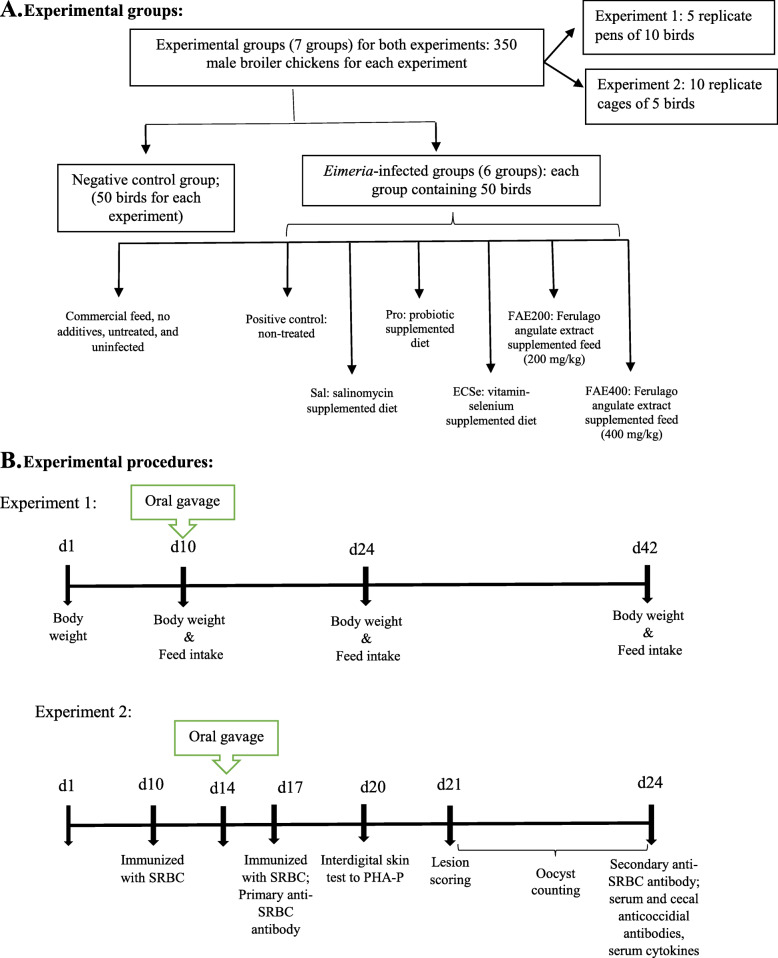
Schematic presentation of the experimental trial. Time values are in days. Chickens were provided with different experimental diets from day 1 until the end of the experiment. All birds except negative control were challenged via oral gavage (1 mL per bird) with sporulated oocysts of *Eimeria* species, including approximately 50,000, 10,000, and 5000 of *E. acervulina, E. maxima*, and *E. tenella* oocysts, respectively [54] on d 10 (for experiment 1) or d 14 (for experiment 2). Birds in the negative control groups were orally provided 1 mL of saline solution, producing the same management stress. Solid arrows (**B**) indicate the days when birds were weighed, bled, and slaughtered for lesion scoring or cecal sampling for determination of *Eimeria*-specific antibody titers. SRBC: sheep red blood cells; PHA-P: phytohemagglutinin-P

All birds except NC were challenged via oral gavage (1 mL per bird) with sporulated oocysts of *Eimeria* species, including approximately 50,000, 10,000, and 5000 of *E. acervulina*, *E. maxima*, and *E. tenella* oocysts, respectively [55] on d 10 (for experiment 1) or d 14 (for experiment 2). These numbers of sporulated oocysts have been proven to have virulence levels necessary to cause infection by the field strains of the chicken coccidian parasites *E. acervulina* [56], *E. maxima* [57], and *E. tenella* [58]. Oocysts of the three species were acquired from the Laboratory of Parasitology, Faculty of Veterinary Medicine, University of Tehran. These field *Eimeria* species’ oocysts were isolated from dropping, litter samples, and intestine of broiler chickens from commercial broiler flocks in Iran. The oocysts were stored in potassium dichromate solution at 28 °C to allow sporulation of the oocysts before storage at 4 °C [59]. For enumeration, oocysts were floated in a saturated salt solution, quantified in a McMaster chamber, and the number of sporulated oocysts were calculated as number of oocysts per mL solution. Sporulated oocysts were then washed free of potassium dichromate and diluted with distilled water to desired concentrations. Birds in the NC groups were orally provided 1 mL of saline solution, producing the same management stress. To avoid coccidia contagious, NC birds were housed separately in identical floor pens (experiment 1) or sterile wire cages (experiment 2) in the same room, with a solid wall partition isolating them from infected birds. All tasks were done with NC chickens first and then with *Eimeria*-infected chickens. Daily checks started with unchallenged birds prior to attending to challenged birds. Manipulations of dietary and *Eimeria* challenge treatments during the entire experimental period is represented in Fig. 7.

### Experiment-1: effect of dietary additives on growth performance parameters

A total of 350 1-d-old male broiler chickens (Ross 308), with a mean initial body weight of 43.1 ± 0.48 g, were randomly allocated to 7 treatments, with 5 replicate pens of 10 birds per pen. The experimental house contained 35 pens of equal size (1.3 m × 1.2 m). Pen body weight and feed intake were recorded at placement, 10, 24, and 42 d of age for calculation of average daily gain (ADG) and average daily feed intake (ADFI) per bird for each replicate pen. Pen body weight was obtained by weighing the total number of birds in each experimental unit. Feed intake was determined as the difference between the amount of feed offered and the amount remaining uneaten during each period. Incidences of mortality were recorded daily in order to determine mortality rate. With the body weight of any deceased or culled chickens included, total ADG and total ADFI for each pen were used for calculating mortality-adjusted feed conversion ratio (FCR) during each feeding period.

### Experiment-2: protective and immunostimulatory effects of dietary additives against coccidiosis

#### Experimental design

On the day of arrival, 350 male broiler chickens (Ross 308), with a mean initial body weight of 42.3 ± 0.57 g, were randomly distributed into 7 treatments having 10 replicate cages of 5 birds per cage (0.30 cm^2^/bird). Broiler chickens were housed in battery cages under thermostatic control with the individual cages serving as experimental units.

#### Coccidiosis lesion scoring

After 7 d of oocyst inoculation (on d 21), 2 broiler chickens of average weight from each cage were euthanized by cervical dislocation for lesion scoring associated with the *Eimeria* challenge. Different intestinal regions for lesion scoring were used because the mixed *Eimeria* species would infect different sections of the intestine simultaneously. All selected birds were lesion scored by the same person who was blinded to the experiment design. Lesions from three sections of the intestine (duodenum, jejunum, and cecum) were ranked from 0 (no gross lesions) to 4 (most severe lesions), according to the standard Johnson and Reid [60] method.

#### Oocyst counting

On d 6–10 post-*Eimeria* inoculation (d 20–24), multiple fresh excreta samples were collected from each cage, pooled, and kept in separate airtight plastic bags. After homogenization, representative samples of 30 g of excreta from each cage were sampled and stored at 4 °C. All samples were processed immediately and after recording the standardized excreta weight (5 g), samples were added in a 50-mL centrifuge tube containing 45 mL of saturated salt solution. After homogenization, excreta samples were loaded in a McMaster counting chamber. Standing at room temperature for 5 min, the oocysts were counted under a microscope and expressed as oocysts per gram of excreta as described by Ghasemi et al. [59].

#### Anti-SRBC antibody titers

To evaluate the primary and secondary antibody-mediated immune response, at 10 and 17 d of age, 2 birds per replicate were immunized intramuscularly with 0.25 mL of 10% sheep red blood cells (SRBC) in phosphate-buffered saline (PBS). At 7 d following each immunization, on d 17 and 24 post-hatching, blood samples (1.5 mL/bird) were collected from the brachial vein of birds and transferred into serum tubes. Blood samples were kept at room temperature for 2 h and then centrifuged at 1500×g for 10 min at 25 °C to isolate serum and stored at − 30 °C for antibody analysis. A direct hemagglutination assay using 96-well, U-bottomed Microtiter plates was performed to determine the total serum antibody response against SRBC. Heat inactivated serum (56 °C for 30 min) was analyzed for total, IgG (mercaptoethanol-resistant), and IgM (the difference between the total and the IgG response) anti-SRBC antibodies according to the procedure described by Cheema et al. [61].

#### Serum *Eimeria*-specific antibody titers

On day 24, 2 birds per cage were randomly selected and blood samples were collected from the wing vein. The blood samples were kept at room temperature (approximately 25 °C) for 2 h and then centrifuged at 580×g for 10 min. *Eimeria*-specific serum antibody levels at d 24 were also measured by in-house ELISA using recombinant coccidia 3-1E protein. It is reported that antibodies produced against the recombinant 3-1E protein reacted with sporozoites and merozoites of *E. acervulina*, *E. maxima*, and *E. tenella* [62]. The 3-1E gene was originally cloned by immunoscreening an *E. acervulina* cDNA library with a rabbit antiserum against *E. acervulina* merozoites [63]. The 1086-base pair 3-1E cDNA was subcloned into the pcDNA expression vector (Invitrogen, Carlsbad, CA), as described [64]. The recombinant 3-lE-pcDNA plasmid was transformed into *E. coli* DH5a and bacteria were grown overnight to the mid-log phase. To purify the recombinant protein, induced *E. coli* cells were harvested by centrifugation and sonicated for 15 min on ice. After centrifugation at 10,000 g, the supernatant was added to a Ni^2+^-nitrilotriacetic acid (Ni-NTA) column (GE Healthcare Life Sciences, Pittsburgh, Pennsylvania, USA) and purified according to the manufacturer’s instructions. An elution buffer (300 mM NaCl, 40 mM Na_3_PO_4_, pH 8.0) containing 500 mM of imidazole was utilized to wash the His-tagged proteins from the Ni-NTA column. The purity of the protein was confirmed by 12% SDS-PAGE and the concentration of the purified protein was determined according to the Bradford procedure [65], using bovine serum albumin as a standard. The purified protein was stored in aliquots at − 70 °C until further use.

To detect anticoccidial antibodies, 96-well round-bottom microtiter plates were coated with 1 μg/well of purified recombinant proteins by overnight incubation at 37 °C. The plates were washed twice with PBS-Tween 20 buffer containing 0.05% Tween-20 (PBS-T), and blocked with PBS containing 1% bovine serum albumin for 1 h at 37 °C. Diluted serum samples (1:50) with the same PBS-T were added into the wells at 100 μl/well, incubated with gentle agitation for 2 h at room temperature, and washed with PBS-T. The wells were then washed 3 times and 100 μl of the following serum polyclonal antibodies was added: horseradish peroxidase (HRP)-conjugated goat anti-chicken IgM (diluted 1:2000), (HRP)-conjugated goat anti-chicken IgA (diluted 1:2000), and alkaline phosphatase-conjugated rabbit anti-chicken IgG Fc fragment specific (diluted 1:10,000). Finally, depending on the secondary antibody used, the colour was developed using 100 μl of a 0.1 μg/ml solution of 2,2′-Azinobis(3-ethylbenzothiazoline-6-sulfonic acid) diammonium salt in citrate buffer (pH 4) (IgA and IgM), or 100 μl of a 1 μg/ml solution of para-Nitrophenylphosphate in diethanolamine (pH 9.8) (IgG) to each well and incubation for 1 h at 37 °C. Bound isotypes in sera or cecal supernatant were quantified by the absorbance at 450 nm by a microplate reader (BioTek, Winooski, VT). All three serum immunoglobulins were within the measurable range in all birds studied.

#### Cecum *Eimeria*-specific antibody titers

On day 24, after blood sampling, cecal contents from 1 bird per cage were removed by flushing the mucosal surfaces with Hank’s balanced salt solution (HBSS; containing 500 IU/mL of penicillin and 500 μg/mL of streptomycin) for tissue culture following the procedures of Zigterman et al. [66]. In brief, Each cecal tissue sample was cut into small pieces (about 2 to 3 mm), washed with HBSS, and suspended in RPMI-1640 modified medium (5 mL), supplemented with gentamicin (100 μg/mL), HEPES buffer (40 mM; pH 7.2), and L-glutamine (2 mM). The suspensions were centrifuged at 300×g for 5 min, and 800-μL aliquots of supernatant were removed at time 0, and then the cells were suspended and incubated at 41 °C, 5% CO_2_, and 95% O_2_ in 25 cm^2^ tissue culture flasks for 16 h. The samples were centrifuged and 800-μL aliquots of supernatant were collected (time 16 h). The specific IgA, IgM, and IgG isotypes in all aliquots (time 0 and 16 h) were determined by an ELISA microplate reader (BioTek, Winooski, VT) as used for serum samples [67]. Cecal antibody levels were calculated by subtracting the A_450_ values of t = 16 aliquots from those of t = 0 ahquots. For each bird, the specific cecal antibody levels were expressed as the mean values (absorbance at 450 nm) of 3 intestinal pieces of 1 g from one cecal tissue.

#### Lymphoproliferation against phytohemagglutinin-P (PHA-P)

**Fig. 8:**
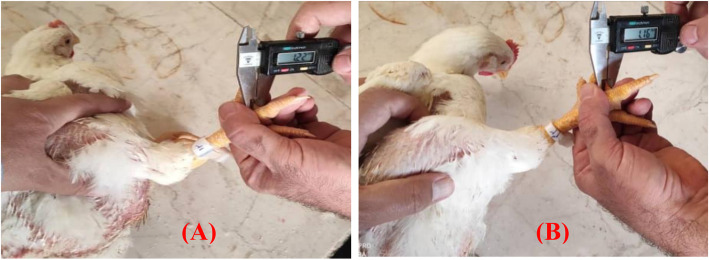
In vivo bioassay for cell-mediated immune response assessed by measuring cutaneous basal hypersensitivity (mm increase in toe web thickness) induced by an intradermal injection of phytohemagglutinin-P between the third and fourth digits of the right foot. Examples: the thickness of the interdigital skin (24 h after injection) from a negative control broiler (**A**) and a positive control broiler (**B)**

Interdigital skin tests to PHA-P for a cell-mediated immune response was conducted in 20-day-old chickens following a previously published procedure [68]. In brief, 100 μg of phytohemagglutinin P (suspended in 0.10 mL of sterile phosphate-buffered saline [PBS]) were injected intradermally between the third and fourth digits of the right foot of 2 birds per cage. The left foot served as a control and was injected with 0.10 mL of PBS. The thickness of the interdigital skin was measured in millimeters with a constant-tension micrometer 24 and 48 h after injection. Lymphoproliferative response to PHA-P was calculated as the change in the thickness of the PHA-injected interdigital site minus the change in thickness of the PBS-injected side. Figure 8 shows the methodology of the cutaneous basal hypersensitivity assay in broiler chickens.

#### Analysis of serum IFN-γ and IL-6

The concentrations of Interleukin-6 (IL-6) and gamma interferon (IFN-γ) in serum samples (*n* = 2 per replicate) were also measured using the commercially available chicken cytokine ELISA kits (Genorise Scientific Inc., Paoli) according to the manufacturer’s instructions). Cytokine concentrations of each serum sample were interpolated from the cytokine standard curve.

#### Statistical analysis

All the data were statistically analyzed as a completely randomized design using GLM procedures using a statistical software computer program (SAS Institute, version 9.0; SAS Institute Inc., Cary, NC, USA) with a pen (experiment 1) or cage (experiment 2) as an experimental unit. The average of 2 birds per replicate cage was applied for parameter analysis of all variables (except oocyst shedding) in experiment 2. Normality and homogeneity of variances were evaluated by Shapiro-Wilk and Levene tests, respectively. Percentage data were transformed to arcsine values prior to analysis if normality was not met. Because the lesion scores were not distributed normally, a one-way non-parametric test (PROC NPAR1WAY and Kruskal–Wallis; SAS Institute, version 9.0; SAS Institute Inc., Cary, NC, USA) was employed. Prior to statistical analysis, log_10_ transformations on oocyst counts and log_2_ transformations were performed on anti-SRBC antibody response. The means were compared using the least squares means procedure (through Proc LSmeans), with Tukey’s adjustment for multiple comparisons at the 0.05 level of probability. All values are expressed as the least square means ± the pooled standard error of the mean (SEM).

## Data Availability

The datasets analysed in the present study are available from the corresponding author upon request.

## Abbreviations

ADFI: Average daily feed intake
ADG: Average daily gain
ECSe: Combination of vitamin E, vitamin C, and selenium
FAE: *Ferulago angulata* extract
FCR: Feed conversion ratio
IFN-γ: Gamma interferon
IL-6: Interleukin-6
NC: Negative control
PBS: Phosphate-buffered saline
PC: Positive control
PHA-P: Phytohemagglutinin P
Pro: Probiotic
Sal: Salinomycin
SRBC: Sheep red blood cell
